# The effects of implementation intentions on prospective memory in young and older adults

**DOI:** 10.3389/fpsyg.2022.905860

**Published:** 2022-06-28

**Authors:** Yu Wen Koo, David L. Neumann, Tamara Ownsworth, David H. K. Shum

**Affiliations:** ^1^School of Applied Psychology, Griffith University, Mt Gravatt, QLD, Australia; ^2^The Hopkins Centre, Menzies Health Institute of Queensland, Griffith University, Gold Coast, QLD, Australia; ^3^Department of Rehabilitation Sciences, The Hong Kong Polytechnic University, Kowloon, Hong Kong SAR, China; ^4^Research Institute of Smart Ageing, The Hong Kong Polytechnic University, Kowloon, Hong Kong SAR, China

**Keywords:** prospective memory, implementation intentions, ageing, older adults, transfer effects

## Abstract

Prospective memory (PM) is the ability to perform a planned action at a future time, while carrying on with other unrelated tasks. Implementation Intentions (II) is a promising metacognitive strategy for improving PM in older adults, though its generalization and longer-term effects are not well-understood. We examined the effects of II on PM in 48 community-dwelling older adults (77% female, *M*_age_ = 71.52) and 59 young adults (75% female, *M*_age_ = 19.86). Participants were randomly allocated to a conventional instruction or II group and administered a laboratory-based PM task in the first session. In the second session, participants returned to complete a similar but new laboratory-based PM task and an ecological PM task without prompts to use a strategy. We found strong age effects on PM performance whereby older adults performed worse than young adults across all PM tasks. While the overall facilitation effect of II was not statistically significant, there was a trend that this strategy facilitated PM performance on the laboratory-based PM task in the first session for older adults with a medium sized effect (*d* = 0.37). The generalization and longer-term effect of II were not significant for either the similar laboratory-based or the ecological PM task. These results suggest that a single-session II intervention may not be sufficient to elicit transfer to other similar new PM tasks in healthy populations.

## Introduction

We often fail to remember to perform an intended action at the right time or on the appropriate occasion, despite every intention to fulfill it. Prospective memory (PM) refers to remembering to carry out an intended action in the future. It includes two components; the prospective component refers to remembering that something must be done, and the retrospective component refers to remembering what needs to be done (Einstein and McDaniel, 1990; Einstein et al., 1995). Successful PM execution depends on retrospective memory processes (e.g., encoding and retrieval), as well as controlled attention and executive processes associated with planning, working memory, and task switching (Kliegel et al., 2002). PM is important for everyday independent functioning in older adults, due to its relevance to quality of life, instrumental activities of daily living, medication management, and maintaining social relationships (McDaniel et al., 2008). Consequently, a large body of literature is now focused on how to maintain and improve PM in later adulthood.

The two main types of PM are time- and event-based (Einstein and McDaniel, 1990; Kvavilashvili et al., 2013). While time-based PM tasks involve remembering to perform an intended action at a specific point in time (e.g., remembering to take medication at 3 PM), event-based PM tasks involve remembering to perform an action when a particular event occurs (e.g., remembering to invite a friend to dinner after an encounter at the café). Event-based tasks can also be focal and non-focal to the ongoing task. Time-based PM tasks typically require individuals to interrupt an ongoing activity to complete the task and require strategic monitoring of time. Meanwhile, event-based PM tasks require individuals to interrupt an ongoing activity when a specific target event occurs. Thus, time-based cues require monitoring of clock or time to perform the intentions at the appropriate time, while event-based cues consist of external events intended to trigger retrieval of PM intention. Both time-based and event-based PM can be the focus of PM training. Overall, there is a difference in resource demands between these event- and time-based tasks, and also based on whether the cue is focal or non-focal.

The multi-process framework (McDaniel and Einstein, 2000; Scullin et al., 2013) posits that successful PM involves either strategic monitoring approach to detect cues in the environment, or spontaneous retrieval (i.e., it just popped into mind). These routes depend on the requirements of the PM task. For example, time-based PM tasks would require strategic monitoring due to higher cognitive resource demands compared to event-based PM, which requires fewer cognitive resources and can rely on spontaneous retrieval. Particularly, when a PM cue is focal to the ongoing task, spontaneous retrieval would suffice. Meanwhile non-focal PM cues would require monitoring. Typically, an interference effect (i.e., slowing of ongoing task response) is observed in non-focal PM tasks and is often absent in focal PM tasks (Einstein and McDaniel, 2005; Brewer et al., 2010; Scullin et al., 2010a,b). This cue focality distinction is relevant due to its theoretical bearings as this distinction dictates which underlying mechanisms supports successful PM (Einstein et al., 2005; McDaniel et al., 2013). That is, due to the higher cognitive demands required for detecting non-focal cues, age effects may be more pronounced in non-focal PM tasks compared to focal PM tasks (Niedzwienska and Barzykowski, 2012).

### Prospective memory training

The concept underlying cognitive training is based on cognitive plasticity— the malleability of behavior (Lövdén et al., 2010). Evidence has suggested that training gains in cognitive performance are still possible in late adulthood (Hering et al., 2014). There are two main approaches to cognitive training— strategy-orientated and process-based training (Reichman et al., 2010). The more common one is strategy training, which is a top-down approach that aims to teach people how to compensate for or circumvent limitations. Meanwhile, process-based training is a restorative, bottom-up approach that aims to augment underlying cognitive processes through repetitive practice. There is an assumption that remediation of core neurocognitive resources should benefit other associated cognitive functions, like planning and intelligence (Morrison and Chein, 2011).

Both strategy and process-based training approaches have been applied to PM. Most PM training studies to date have investigated clinical samples with PM deficits, such as those with traumatic brain injury and mild cognitive impairment (Fleming et al., 2005; Shum et al., 2011; Fish et al., 2015). For instance, a randomized control trial for individuals with traumatic brain injury by Shum et al. (2011) included four intervention groups which consisted of: (1) self-awareness plus compensatory PM training; (2) self-awareness training plus active control; (3) active control plus compensatory PM training; and (4) active control only. Active control for self-awareness training involved psychoeducation about attention, concentration, brain function through documentary material without reference to PM. Compensatory PM training involved rehabilitation training strategies to compensate for PM problems. Lastly, self-awareness training facilitated insight into PM problems and its relevance to daily life. All training groups were assessed on a standardized PM test and self-reported PM measures. Following the 8-session individual intervention programs, the compensatory training group showed larger pre-test to post-test change scores for the standardized PM test. Meanwhile there were no training effects for the remedial training conditions. Furthermore, training gains did not differ between the groups with or without the self-awareness training. Rose et al. (2015) compared the effectiveness of computer-based PM training based on Virtual Week (a computerized board game simulating every-day tasks) with both active and passive control groups among 59 healthy older adults (*M*_*age*_ = 67.4; *SD* = 4.77). The training group received process-based training through the computerized task over 4 weeks (three sessions a week, and two levels per session). Meanwhile the active control group received computerized training on basic musical concepts including rhythm, pitch, melody and voice through a combination of motor, perceptual and cognitive tasks over the 4 weeks. The control group only completed the baseline measures at pre-test and post-test. The training group showed gains on the Virtual Week game, and transfer effects to a call-back task. However, no gains were observed on the laboratory event-based task and the Cooking Breakfast task. Although there is evidence that PM interventions are able to produce training gains, there are currently only 11 studies on PM interventions among healthy older adults; and nearly half of these studies included PM training as part of a larger memory training program (Tsang et al., 2021).

### Generalization of cognitive strategies

An important concept in cognitive training is transferability. This is the degree to which a learned skill generalizes to other contexts/untrained tasks (Lövdén et al., 2010). Training and transfer context effects can be categorized as near-transfer and far-transfer, which refers to the generalization of skill learned to proximal or more distant domains, respectively (Zelinski, 2009). Near-transfer relates to when the training task and subsequent untrained task are tapping into the same underlying cognitive processes. For example, training on one type of working memory task and subsequently showing improvement (i.e., gains) in a similar, yet distinct working memory task reflects near-transfer. Far-transfer occurs when training on a specific task produces generalized improvement in performance on a task that has little or no overlap with the training task. For example, the finding that training in auditory working memory also improves performance on an auditory processing task would represent far-transfer. Although there is ample evidence showing that task-specific improvement is possible after PM training, few studies have investigated near and far-transfer effects, possibly due to a lack of standardization in categorizing transfer tasks as either near or far (Buitenweg et al., 2012).

Far-transfer can also occur when skills transfer from a training context to everyday functioning, which may be reflected by a reduction in problems with instrumental activities of daily living (Willis et al., 2006). However, research on transfer effects following cognitive training in the older population is lacking, especially for PM training (Zelinski, 2009). Process-based training is often criticized for lack of transfer to everyday tasks (Craik and Rose, 2012; McDaniel and Bugg, 2012). Yet, strategy-orientated training gains are mostly limited to the training task itself (Hering et al., 2014). Thus far, evidence of far-transfer effects for both strategy and process-based training approaches are limited, and these criticisms also apply to PM (Hering et al., 2014).

### Implementation intentions

A strategy to improve the likelihood of performing an intended action is by translating goal intentions into an implementation intention (Gollwitzer, 1999). Implementation Intentions (II) is a metacognitive strategy that requires an individual to specify when, where, and how to act on a given goal following an “if-then” format: If I encounter X situation, then I will initiate action Y (Gollwitzer, 1999; Gollwitzer and Sheeran, 2006). Its effectiveness has been demonstrated across a variety of tasks, from breast self-examination (Luszczynska and Schwarzer, 2003), physical training (Sniehotta et al., 2006), healthy eating (Verplanken and Faes, 1999), and in experimental go no-go paradigms (Brandstätter et al., 2001). A meta-analysis including 94 studies found a medium to large effect size for improving goal attainment following training in II (*d* = 0.61; Gollwitzer and Sheeran, 2006).

Previous studies have shown that II can enhance PM function in younger adults (*d* = 0.45; Chen et al., 2015). More recently, this has been extended to healthy older adults in both laboratory (Chasteen et al., 2001; McFarland and Glisky, 2011; Henry et al., 2020) and naturalistic settings (Liu and Park, 2004; Burkard et al., 2014a). However, not all studies have reported positive benefits of II on PM in healthy older adults (Schnitzspahn and Kliegel, 2009). Some studies have also distinguished the differences for this effect between event- versus time-based tasks and found inconsistent effects (Mioni et al., 2015; Foster et al., 2017; Liu et al., 2018; Henry et al., 2020). These findings suggest that II should be more effective for event-based tasks because it is easier to generate an if-then associative link for an environmental cue. Meanwhile for time-based tasks (i.e., time-monitoring) environmental cues are crucial for completing the intended action. Furthermore, evidence suggests the benefits are greater for older adults with low fluid intelligence (Brom et al., 2014) or limited executive abilities (Brom and Kliegel, 2014). Meanwhile, some studies have found no relation between executive functions/working memory and the effectiveness of II (McFarland and Glisky, 2011; Burkard et al., 2014b). Thus far, the positive effects of II on PM have mostly been observed in clinical populations, such as people with multiple sclerosis (Kardiasmenos et al., 2008), brain injuries (Grilli and McFarland, 2011), schizotypal features (Chen et al., 2014), and autism spectrum disorder (Kretschmer-Trendowicz et al., 2016).

Although forming II is a conscious act, the retrieval mechanisms are assumed to be automatic or non-conscious, rather than deliberate. The two assumptions underlying this facilitation effect are that: (1) II should improve encoding of intentions, which reduces the need for monitoring and thus results in more automatic detection of PM cue and facilitates spontaneous retrieval of intention (McDaniel and Scullin, 2010), and (2) this strategy strengthens the perceived importance of the intention and may allocate more resources to monitoring in the context of the ongoing activity (Meeks and Marsh, 2010). Theoretically, this strengthens the cue-response link, and so when the target cue occurs (e.g., walking past grocery store), the intended action (e.g., buying bread) is spontaneously retrieved and executed. Thus, according to the multi-process theory, II should facilitate performance for tasks that require strategic monitoring (i.e., non-focal event-based tasks, time-based tasks).

This strategy can be encoded in a verbal form (repeating instructions), imagery form (imagining relevant scenes) or both forms combined. Evidence suggests that for older adults, there is an additive effect when using both forms combined for event- but not time-based PM tasks (Henry et al., 2020). Brom and Kliegel (2014) compared a process/restorative approach of executive control (task-switching) and II (strategy training) in 62 community-dwelling older adults. Effects were examined on an ecological PM task involving blood pressure monitoring. To assess near and far-transfer of task-switching training to related and other cognitive domains, a set of cognitive tasks were used (processing speed, short-term memory capacity). Participants were randomly allocated to: (1) combined process training/strategy training, (2) process training/no strategy training), (3) no process training/II strategy training), or (4) no process training/no strategy training. Older adults in the strategy training group outperformed the process training group in frequency and accuracy on the blood pressure task. Although no transfer effect was observed on untrained cognitive tasks, they found that II significantly facilitated PM performance for participants with low task-switching ability.

Consequently, Henry et al. (2021) conducted a randomized control trial comparing PM performance in healthy older adults (60–87 years old) using four, 6-week interventions: (1) restorative or process-based (*N* = 30), directly training PM, (2) compensatory training involving psychoeducation, II, monitoring strategies, and external reminders (*N* = 31), (3) combined restorative and compensatory (*N* = 31), and (4) active control (*N* = 32). They investigated near-transfer to the same PM tasks, and far-transfer effects to untrained cognitive domains and functional capacity on the Timed version of Instrumental Activities of Daily Living task. They found that only the combined condition involving restorative and compensatory approaches led to post-training improvements (transfer effects) on the PM tasks. No far-transfer effects were evident for any intervention. Due to their multi-component intervention approaches, the specific training effects of II could not be established.

While a growing body of research has examined the efficacy of II for improving PM in older adults, most studies have not investigated short-term transfer effects of this strategy on a subsequent similar untrained task (Chasteen et al., 2001; Schnitzspahn and Kliegel, 2009; Zimmermann and Meier, 2010; McFarland and Glisky, 2011; Henry et al., 2020). A systematic review and meta-analysis investigating the effect of II on PM revealed a medium to large effect size (*d* = 0.68) for older adults, although this was not homogenous across older adults due to heterogeneity of sample groups (Chen et al., 2015). The authors speculated that this may be due to a large age range and different II encoding strategies used in the analyzed studies (Chen et al., 2015). Thus far, evidence suggests that the beneficial effects of a single session II involving combined visual and verbal strategies are relatively consistent and robust among healthy older adults, particularly for event-based PM (Chen et al., 2015). Importantly, the transfer of this facilitation effect after a single training session to other non-trained PM tasks or task performance after a delay has yet to be examined. Moreover, there is a lack of uniformity in categorizing transfer tasks as either near or far (Buitenweg et al., 2012). Lastly, in most studies, the conventional and II conditions are not tightly matched in instructions, with community-based studies not allowing control over intervening tasks during daily life. Consequently, the effect of II on PM performance may have been overestimated.

### Rationale and aims of the current study

This study aimed to investigate the effect of an II strategy on event-based PM, and its transfer effects for healthy young and older adults. As a novel extension, to assess the transferability of II, we introduced a 7-day lag between the first and second PM tasks which were conducted in a controlled (laboratory) environment rather than a community context (e.g., Brom et al., 2014).

Despite a meta-analysis identifying medium to large effect sizes (Chen et al., 2015), it is important to examine whether the benefits of this strategy extend or transfer beyond training after a single-session implementation. To assess near-transfer, we introduced a similar but different laboratory-based task. For far-transfer, we adapted an ecological task which embedded both time- and event-based PM from Shum et al. (2013). This involved a recipe task that was complex but still familiar to both age groups, while also being conducted in a controlled environment.

Assuming that the effects of II are robust, it was hypothesized that: (1) The II groups would perform significantly better immediately following training on the first laboratory-based PM task compared to the control condition (conventional instructions) for both older and younger adults; (2) a near-transfer effect would be observed in terms of improved performance on the second laboratory task for those in the II group; and lastly (3) a far-transfer effect would be observed on the untrained ecological PM task, with those in the II group performing better than those in the control group.

## Methods

### Participants

An *a* priori power analysis using G^*^Power (Faul et al., 2009) indicated that a total sample size of 124 (62 young adults, 62 older adults) was required to detect a medium effect (*f* = 0.25), with an alpha probability of 0.05 and power of 0.80. In the end, a total of 119 participants were recruited for this study [61 young adults (*M*_age_ = 19.77 years, *SD* = 3.69, 73.8% females), and 58 older adults (*M*_age_ = 71.84 years, *SD* = 5.96, 75.9% females)]. All participants were native English speakers, had normal or corrected-to-normal vision, and had no history of neurological or psychiatric disorders. Young adults (age range: 17–32 years) were undergraduate university students who were compensated with course credit. Healthy older adults (age range: 63–90 years) were recruited from the general community. The initial screening process was conducted over telephone using the Telephone Interview for Cognitive Status (TICS-M; Brandt et al., 1988). Inclusion criteria for older adults were TICS-M score ≥ 31 (Knopman, 2010), Mini-Mental State Examination (MMSE) score ≥ 25 (Woods et al., 2014), no history of neurological illness or brain injury, no current or history of major psychiatric illness, no current or history of alcohol or substance abuse, and no significant visual or hearing impairment. Participants who did not perform any intended PM actions and were unable to recall the PM instructions post-experiment were excluded from all analyses (2 young and 10 older adults), as this reflects retrospective memory rather than PM failure (Zimmermann and Meier, 2006; Heathcote et al., 2015).

### Design

The two between-participants factors are age (young vs. old) and instructions (conventional vs. II). Participants were randomly allocated to either the conventional (CI) or II group. The dependent variables were PM task accuracy (%), ongoing task accuracy, and reaction time (RT) for each task.

### Measures

#### Cognitive measures

##### Telephone interview for cognitive status modified (TICS-M)

The TICS-M (Welsh et al., 1993) was used to screen older adults over the telephone. The TICS-M is a brief 13-item test of cognitive functioning with scores ranging from 0 to 50. TICS-M is as reliable and valid as face-to-face administration and has a sensitivity of 94% and specificity of 100% for distinguishing healthy older adults from individuals with dementia (Brandt et al., 1988), and those with mild cognitive impairment (Knopman, 2010).

##### Mini-mental state examination (MMSE)

MMSE is a measure of general cognitive functioning commonly used in older adults (Folstein et al., 1975). This scale includes 11 questions and requires 5–10 min to administer. It focuses on the cognitive aspects of mental functions and is divided into two sections. Scores range from 0 to 30. A score of 24 and higher indicates that individuals are cognitively intact, meanwhile scores of 23 and lower are indicative of cognitive impairment.

##### Wechsler abbreviated scale of intelligence (WASI-II)

The WASI-II is a short form IQ test designed to measure intelligence and cognitive ability in adults and older adolescents (ages 6–89 years). In this study, the two subscales vocabulary and matrix reasoning were used to calculate FISQ-2. In an adult sample, the average internal consistency reliability coefficients for FSIQ-2 is 0.96 with test re-test reliability of 0.88.

##### Letter-number sequencing subtest (LNS)

The LNS is a subtest of the Wechsler Memory Scale–III (Wechsler, 1997) that measures working memory. Participants were orally presented with a series of alternating numbers and letters, and asked to recall the numbers in numerical order followed by the letters in alphabetical order. The test begins with series of two items (one number and one letter) and continues to a maximum of eight items (four numbers and four letters). Participants were given three trials at each series length and continued until all three trials of a series length are failed. The maximum possible score for LNS is 21.

#### PM tasks

##### The silly sentences task (SST)

The SST was administered as a measure of immediate post-training PM performance. For the ongoing task, participants were required to read 100 sentences and respond “true” (e.g., Apples are a fruit), or “false” (e.g., Apples have loud voices) as quickly as possible whilst avoiding errors (press “F” key for true, and “J” key for false). Sentences were developed based on the Silly Sentences Test, an instrument measuring speed of language comprehension (Baddeley et al., 1992) that is similar to previous PM tasks (Blanco-Campal et al., 2009; Fish et al., 2015). There was a total of 142 sentences with 12 non-focal PM cues. For the PM task, participants were asked to press “K” when a red border appeared around the sentences. The PM cue was a red border around the sentence and appeared at pseudorandom intervals (i.e., 13, 21, 40, 50, 53, 61, 81, 88, 97, 109, 114, 140). For each trial, the border colors were either red, blue, cyan, purple, orange, yellow, pink, green, white, or gray. For each trial, a fixation cross was presented in the center of the screen for 1,000 ms followed by a sentence presented for 3,000 ms. The outcome measures were PM task accuracy (percentage correct), ongoing task accuracy (percentage correct) and reaction time. Pilot testing revealed that stimulus presentation times were optimal to prevent ceiling effects for young adults, and also adequate to prevent floor effects for older adults.

##### Lexical decision task (LDT)

This task was developed based on Einstein and McDaniel (1990)'s dual-task paradigm for the near-transfer task, and adapted from Hogan et al. (2021). Participants were presented with letter strings, and participants judged whether they are words or non-words as the ongoing task (pressing “F” for words, and “J” for non-words). Each string consisted of four letters and were presented in capitals in a clear legible font. words were sourced from the MRC Psycholinguistic Database (Wilson, 1988) and legal non-words from the ARC Nonword Database (Rastle et al., 2002). For words, the mean age of acquisition rating was 323 (i.e., between age 5 and 6), with a mix of low frequency (*N* = 42), medium frequency (*N* = 19) and high frequency words (*N* = 29), and words without frequency ratings (*N* = 7). There were 206 words (97 words, 97 non-words) and 12 non-focal PM cues. For the PM task, participants were asked to press “K” when a red border appeared around the strings. The PM cue was a red border around the word and appeared at pseudorandom intervals (i.e., trials 15, 22, 29, 36, 50, 107, 121, 132, 161, 168, 183, 187). For each trial, the border colors were either red, blue, cyan, purple, orange, yellow, pink, green, white, or gray. For each trial, a fixation cross was presented in the center of the screen for 1,000 ms, followed by a stimulus which could either be a word, a non-word, or a PM cue displayed for 1,000 ms. The outcome measures were PM task accuracy (percentage correct), ongoing task accuracy (percentage correct) and reaction time.

##### Ecological PM task

The ecological PM task was adapted from Shum et al. (2013) and included event-based and time-based components simultaneously. This task was included as the far-transfer task. For the ongoing task, participants were instructed to sit at their own kitchen table or a mock kitchen table (see Procedure) to use a recipe book containing 10 recipes and asked to calculate the total cost of each recipe using a grocery catalog, working from the first page to the last page. The number of items per recipe ranged from 6 to 11, and each recipe was a page long. For the event-based PM task which was a focal cue, participants were to bookmark recipes with a sticky note that are free from dairy, eggs, and meat (including fish). The explanation for bookmarking dairy-free recipes was that one of the guests coming to dinner may be allergic to dairy products. Four of these targets were placed at fixed intervals through the book. A proportion correct score was calculated based on the number of correct recipes bookmarked. For example, if three were correctly marked, a score of 75% was awarded. If the participant turned the page and moved on without bookmarking a target recipe, it was marked as missed due to no action being carried out. The maximum score for this task is 100%.

The time-based PM task was non-focal and required participants to check the computer tablet (swipe up, to unlock) at certain time intervals using a kitchen timer placed slightly to the side just out of direct vision of the participant. The timer counted from 0:00 until the end of the task. Participants were to unlock the tablet at 8 min, and then every 7 min after that (15, 22, 29, etc.) until the task was completed. Participants were given these instructions at the beginning of this task. Those who carried out the PM action within 15 s before or after the expected time were scored as a “hit” and those who carried out the action outside of that 15 s window were scored as “missed.” The number of time-based tasks correctly performed were scored as proportion correct as this naturalistic task length varies for each individual. For example, if a participant took 21 min to complete the task, there were 3 time-based PM cues. The maximum score for this task is 100%. The average completion time was 33 min (range: 31–35 min) and 43 min (range: 40–47 min) for young and older adults, respectively.

### Procedure

To enhance the feasibility of recruiting the older adult population, they were assessed in their own homes if requested (98% did so), while all young adults participated on campus. All participants gave their written consent to participate, and the study was approved by the institution ethics committee. All participants were randomly allocated to either CI or II condition. The two groups received the same assessment package with the only difference being the instructions to the Silly Sentences Task (SST) in their first session. In Session 1, testing commenced with the SST, followed by the cognitive measures (i.e., MMSE, WASI-II and LNS) which were collected for another study. At the end of session 1, all participants were told “Thank you for attending this session. What we asked you to do today is something people use to help them to remember things. If you find this useful, you can try using it in your everyday life.” In session 2, the recipe task and LDT were administered in counterbalanced order. No prompting on strategy use was provided on the LDT or recipe task for any participants.

Those in the II condition were given instructions to write down specific information combined to form the “if-then” implementation intention statement (e.g., “If I see a red border in the task, then I will press K”). They then (a) spoke the if-then sentence three times aloud, (b) wrote the statement thrice on a sheet of paper and finally, (c) memorized the sentence and repeated it once to the experimenter, and (d) visualized the specific circumstances for when and where they will carry out the intended PM action.

The CI group followed the same encoding protocol, except their instruction statement was: I will press the Press the K key when there is a red border, rather than the “If-Then” statement which is the key component of II. To ensure equal exposure and rehearsal, participants were instructed to (a) speak the sentence three times aloud; (b) copy down the intention thrice and; (c) repeat the memorized sentence once to the experimenter; and (d) visualize walking on a beach. This procedure was designed to ensure that the effect of II is evaluated against a standardized and comparable instruction condition. They were then asked to complete self-reported measures during a 5-min interval prior to completing the PM tasks and cognitive battery.

All participants were required to complete their second session of testing in 7 days. Session 1 and 2 took ~1.5 h each. All participants were asked to describe the requirements of the PM tasks to ensure their understanding, and were also asked to recall instructions for ongoing and PM tasks at the end of both sessions.

#### Statistical analyses

All data were analyzed using SPSS 25. Inferential analyses were conducted using independent samples *t*-test to investigate differences between CI and II groups as well as between age groups on demographic and cognitive variables. Between-group differences in PM performance were examined using 2 (age group: young vs. older) × 2 (condition: CI vs. II) between-subjects ANOVA, with significant ANOVAs followed up by planned comparisons (one-tailed tests). For all statistical analyses α was set at 0.05.

## Results

Data from 48 neurologically healthy older adults (37 women, 11 men, 94.8% Caucasian, *M*_age_ = 71.52, *SD* = 5.57) and 59 healthy young adults (44 females, 15 males, 83.6% Caucasian, *M*_age_ = 19.86, *SD* = 3.72) were analyzed for the current study. Two young adults and 10 older adults were excluded from analyses due to failure to score at least 50% correct for the ongoing tasks on the LDT and SST. No older adults were excluded based on cognitive screening scores indicating that all participants were cognitively healthy. However, older adults had significantly higher years of education, *t*_(61.04)_ = −2.51, *p* = 0.015, *d* = −0.52, working memory, *t*_(105)_ = −5.78, *p* = 0.000, *d* = −1.12 and IQ, *t*_(105)_ = −3.76, *p* = <0.001, *d* = −0.93 than young adults (see Table 1 for demographic variables across age group and conditions). Distributions of data across all variables of interest were inspected visually using QQ plots for univariate outliers, significant skewness. Univariate outliers were defined as those with *Z*-scores greater than ±3; and significant skewness/kurtosis was defined as degree of skewness/kurtosis divided by standard error of skewness/kurtosis being greater than ± 3 (Tabachnick et al., 2007). All variables followed a normal distribution. There were no missing data.

**Table 1:** Demographic characteristics and cognitive measures by age group and condition.

	**Young adults**				**Older adults**			
	**CI (*****n*** **= 29)**		**II (*****n*** **= 30)**		**CI (*****n*** **= 22)**		**II (*****n*** **= 26)**	
	***M***	***SD***	***M***	***SD***	***M***	***SD***	***M***	***SD***
Age	20.10	3.35	19.63	4.09	71.68	5.64	71.38	5.62
TICS-M					34.77	3.09	33.65	2.62
MMSE					29.73	0.46	29.58	0.81
Education (years)	12.69	1.54	12.63	1.50	14.09	3.88	13.96	3.23
LNS	10.07	2.69	9.17	1.86	12.00	2.18	12.46	2.55
FSIQ-2	102.31	10.34	101.13	9.79	108.50	11.68	110.65	12.26

*CI, Conventional Instructions; II, Implementation Intentions; TICS-M, The Telephone Interview for Cognitive Status modified; MMSE, Mini-Mental State Examination; FSIQ-2, Full Scale Intellectual Quotient from Wechsler Abbreviated Scale of Intelligence-II; LNS, Letter Number Sequencing*.

### Session 1

#### Direct effect of II on PM (SST)

A 2 (age group: young vs. older) × 2 (condition: CI vs II) between-subjects ANOVA was conducted for SST PM accuracy. There was a significant main effect of age group, *F*_(1, 103)_ = 51.24, *p* < 0.001, ${\eta_{p}}^{2}=$ 0.332, with older adults performing poorer (*M* = 0.57, *SD* = 0.29) compared to young adults (*M* = 0.87, *SD* = 0.15). There was no main effect of condition, *F*_(1, 103)_ = 1.84, *p* = 0.178, ${\eta_{p}}^{2}$ = 0.018, and no significant interaction for age group × condition, *F*_(1, 103)_ = 1.25, *p* = 0.267, ${\eta_{p}}^{2}=$ 0.012.

A 2 (age group: young vs. older) × 2 (condition: CI vs. II) between-subjects ANOVA was conducted for SST OT accuracy. There was a main effect of age group, *F*_(1, 103)_ = 49.49, *p* < 0.001, ${\eta_{p}}^{2}$ = 0.325, with older adults performing poorer (*M* = 0.73, *SD* = 0.12) compared to young adults (*M* = 0.86, *SD* = 0.07). There was no main effect of condition, *F*_(1, 103)_ = 1.05, *p* = 0.308, ${\eta_{p}}^{2}$ = 0.010, and no significant interaction for age group × condition, *F*_(1, 103)_ = 0.43, *p* = 0.514, ${\eta_{p}}^{2}$ = 0.004.

See Table 2 for mean scores on all PM and ongoing tasks. Guided by theoretical assumptions, planned comparisons were conducted comparing PM accuracy between conditions by age group using one-tailed *t*-tests. For young adults, there were no significant differences between CI and II conditions, *t*_(47.39)_ = 0.26, *p* = 0.40, *d* = 0.07. For older adults, there was a trend for those in the II condition (*M* = 0.62, *SD* = 0.22) to perform better than those in the CI condition (*M* = 0.51, *SD* = 0.36), although this was not significant, *t*_(33.39)_ = 1.24, *p* = 0.11, *d* = 0.37. Bayes Factors was also conducted to further explore this trend which revealed that, BF_10_ = 2.22, 95% credible intervals: −0.07 - 0.29, and thus that the data provides anecdotal evidence for H1.

**Table 2:** Proportion of correct responses (%) on PM tasks and ongoing tasks and completion time.

	**Young**				**Old**			
	**CI (*****n*** **= 29)**		**II (*****n*** **= 30)**		**CI (*****n*** **= 22)**		**II (*****n*** **= 26)**	
	***M***	***SD***	***M***	***SD***	***M***	***SD***	***M***	***SD***
**SST**								
Ongoing task	0.88	0.05	0.84	0.09	0.73	0.13	0.72	0.12
PM accuracy	0.87	0.18	0.88	0.12	0.51	0.36	0.62	0.22
**LDT**								
OT accuracy	0.87	0.05	0.86	0.07	0.79	0.09	0.79	0.10
PM accuracy	0.77	0.16	0.79	0.16	0.45	0.27	0.49	0.21
**Recipe task**								
Completion time (mins)	32:32	0:55	34:38	1:32	42:10	2:14	45:06	2:23
OT accuracy	0.73	0.15	0.78	0.15	0.70	0.13	0.71	0.10
Event-based PM	0.86	0.16	0.87	0.16	0.64	0.34	0.64	0.39
Time-based PM	0.78	0.28	0.86	0.24	0.62	0.37	0.64	0.30

*CI, Conventional Instructions; II, Implementation Intentions; SST, Silly Sentences Task; LDT, Lexical Decision Task; PM, Prospective Memory; OT, Ongoing Task*.

### Session 2

#### Near-transfer effect of II on PM (LDT)

A 2 (age group: young vs. older) × 2 (condition: CI vs. II) between-subjects ANOVA was conducted for LDT PM performance. There was a significant main effect of age group, *F*_(1, 101)_ = 77.00, *p* < 0.001, ${\eta_{p}}^{2}$ = 0.433, with older adults performing worse (*M* = 0.47, *SD* = 0.24) than young adults (*M* = 0.80, *SD* = 0.13). There was no main effect of condition, *F*_(1, 101)_ = 0.93, *p* = 0.337, ${\eta_{p}}^{2}$ = 0.009, and no significant interaction between age group × condition, *F*_(1, 101)_ = 0.05, *p* = 0.830, ${\eta_{p}}^{2}$ = 0.000. Planned comparisons for PM accuracy between conditions by age group revealed that for young adults, there were no significant differences between CI and II conditions, *t*_(56.75)_ = 0.53, *p* = 0.30, *d* = 0.13. For older adults, there was also no significant differences between CI and II conditions, *t*_(39.21)_ = 0.62, *p* = 0.27, *d* = 0.17.

A 2 (age group: young vs. older) × 2 (condition: CI vs. II) between-subjects ANOVA was conducted for LDT OT accuracy. There was a significant main effect of age group, *F*_(1, 103)_ = 26.02, *p* < 0.001, ${\eta_{p}}^{2}$ = 0.202, with older adults performing poorer (*M* = 0.79, *SD* = 0.09) compared to young adults (*M* = 0.86, *SD* = 0.06). There was no main effect of condition, *F*_(1, 103)_ = 0.28, *p* = 0.596, ${\eta_{p}}^{2}$ = 0.003, and no significant interaction between age group × condition, *F*_(1, 103)_ = 0.05, *p* = 0.825, ${\eta_{p}}^{2}$ = 0.004.

#### Far-transfer effect of II on PM (Ecological)

##### Event-based task

A 2 (age group: young vs. older) × 2 (condition: CI vs. II) between-subjects ANOVA revealed a significant main effect of age group, *F*_(1, 100)_ = 16.86 *p* = <0.001, ${\eta_{p}}^{2}$ = 0.14, whereby young adults scored significantly higher (*M* = 0.86, *SD* = 0.16) compared to older adults (*M* = 0.64, *SD* = 0.36). However, there was no main effect of condition, *F*_(1, 100)_ = 0.00, *p* = 0.944, ${\eta_{\text{p}}}^{2}\;$ = 0.000, and no significant condition × age group interaction, *F*_(1, 100)_ = 0.01, *p* = 0.924, ${\eta_{\text{p}}}^{2}$ = 0.000. Planned comparisons for event-based PM accuracy between conditions by age group revealed that for young adults, there were no significant differences between CI and II conditions, *t*_(52.19)_ = 0.44, *p* = 0.33, *d* = 0.10. For older adults, there was also no significant differences between CI and II conditions, *t*_(45.39)_ = 0.62, *p* = 0.46, *d* = 0.00.

##### Time-based task

**Figure 1:**
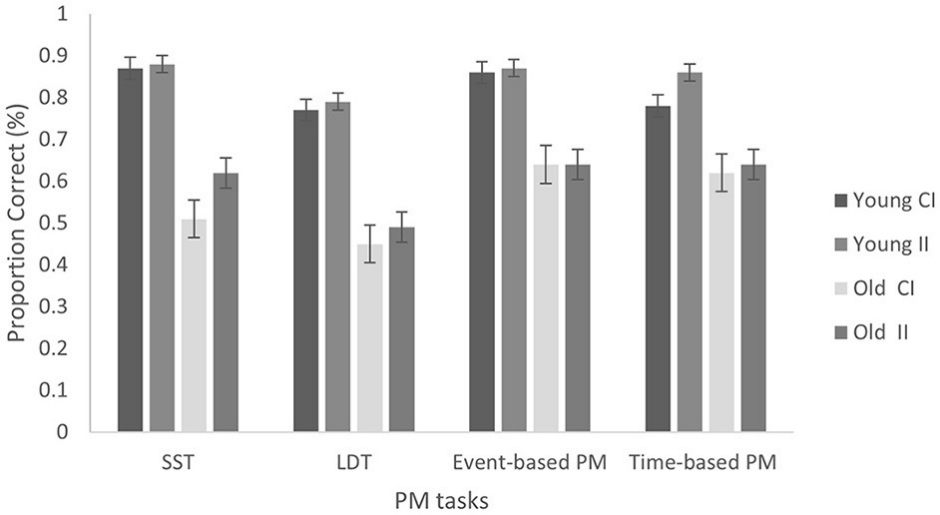
PM performance in all PM tasks for both age groups and conditions. Error bars represent the standard error (SE). CI, Conventional Instructions; II, Implementation Intentions.

A 2 (age group: young vs. older) × 2 (condition: CI vs. II) between-subjects ANOVA revealed a significant main effect of age group, *F*_(1, 98)_ = 9.15, *p* = 0.004, ${\eta_{p}}^{2}$ = 0.085, whereby young adults scored significantly higher (*M* = 0.82, *SD* = 0.26) compared to older adults (*M* = 0.63, *SD* = 0.33). However, there was no main effect of condition, *F*_(1, 98)_ = 0.61, *p* = 0.438, ${\eta_{p}}^{2}$ = 0.006, and no significant condition × age group interaction, *F*_(1, 98)_ = 0.32, *p* = 0.576, ${\eta_{p}}^{2}$ = 0.001. The proportion of correct PM responses across the age groups and instruction conditions in the all PM tasks are presented in Figure 1. Planned comparisons for time-based PM accuracy between conditions by age group revealed that for young adults, there were no significant differences between CI and II conditions, *t*_(103)_ = 1.01, *p* = 0.16, *d* = 0.27. For older adults, there was also no significant differences between CI and II conditions, *t*_(103)_ = 0.30, *p* = 0.38, *d* = 0.09.

##### Recipe ongoing task

A 2 (age group: young vs. older) × 2 (condition: CI vs. II) between-subjects ANOVA was conducted for OT accuracy. There was a significant main effect of age group, *F*_(1, 102)_ = 2.79, *p* = 0.098, ${\eta_{p}}^{2}$ = 0.027, with older adults performing poorer (*M* = 0.71, *SD* = 0.14) compared to young adults (*M* = 0.75, *SD* = 0.15). There was no main effect of condition, *F*_(1, 102)_ = 1.00, *p* = 0.320, ${\eta_{p}}^{2}$ = 0.010, and no significant interaction between age group × condition, *F*_(1, 102)_ = 0.33, *p* = 0.565, ${\eta_{p}}^{2}$ = 0.004^1^.

## Discussion

The aim of this study was to investigate the training and transfer effects of II on PM tasks across older and younger adults. Overall, we found strong age effects across all PM tasks, but no significant effects of II or transfer effects across the tasks. Contrary to expectations there were no significant differences in PM performance between II and CI groups in either age groups for any of the three PM tasks. While not statistically significant, there was a trend with a medium effect sized effect whereby older adults in the II group performed better than the CI group on the first laboratory-based task. This trend was not observed for young adults. Older adults also performed similarly across the event- and time-based naturalistic PM task, with age effects larger in magnitude for the event-based PM task.

### Aging and PM

Interestingly, despite the older adults having significantly higher years of education and better working memory and IQ than young adults, age-related differences in PM performance were consistent and robust across all three PM tasks. However, this is somewhat expected, since cognitive processes such as learning, memory and executive functions that rely on the prefrontal and medial temporal cortex functions show considerable decline with age (Fisk and Sharp, 2004; Burke and Barnes, 2006; Luo and Craik, 2008). In addition, PM is heavily reliant on both prefrontal systems (Brodmann Area 10; Burgess et al., 2001) and the medial temporal lobe (Gordon et al., 2011), hence, strong age effects are expected when comparing PM performance on tasks that involve these cognitive functions. Although there is evidence suggesting that older adults' naturalistic PM abilities are preserved due to the routine nature of tasks (i.e., instrumental daily activities) which rely on more automatic cognitive processes than attentional and executive functioning (McDaniel and Einstein, 2007), this was not supported by the current results for the ecological PM task. Thus, PM should be routinely assessed for older adults along with other cognitive abilities due to its incremental importance in everyday functioning (Sheppard et al., 2020).

### II and prospective memory

Despite no significant effects of II on any PM task, the trend level findings for the SST PM task were in the predicted direction with a medium effect size. That is, the older II group performed better than the older CI group on the SST PM task, while no such trend was observed for the younger adults' conditions. This is consistent with the literature that II may facilitate performance, although the robustness and parameters of the strategy are still in question. For example, Schnitzspahn and Kliegel (2009) found mixed results whereby young-olds (aged 60–75-years) benefited while older-olds (aged 76–90-years) did not. Moreover, detrimental effects (i.e., worse performance) were observed in the older-olds implementing this strategy. It is important to note that studies using this encoding strategy observed these effects in clinical and subclinical populations involving, for instance, individuals with low fluid intelligence (Brom et al., 2014) or limited executive abilities (Brom and Kliegel, 2014). Our findings that II did not significantly facilitate PM performance on the first PM task may be a result of the high functioning (higher IQ, education) older adult sample in this study, although controlling for these variables did not affect PM task performances either.

Alternatively, the lack of facilitation effects may be due to the well-matched instructions of both CI and II groups. We found that for older adults, the effect size (*d* = 0.37) for II is weaker than Chen et al. (2015)'s findings (*d* = 0.68). In this study, we rigorously ensured the comparability of instructions except for the if-then link, and the visualization component. In both conditions, participants repeated the instructions three times and wrote them down three times. The only difference was that participants in the II group imagined carrying out the intention, while the CI group imagined walking on a beach. Other studies have typically not controlled for the visualization part of their instructions or matched them in terms of multi-modal components (e.g., Brom et al., 2014; Henry et al., 2020). For example, Brom et al. (2014) had control participants read about blood pressure-related issues while the II group visualized their task intentions. We cannot rule out that participants truly visualized walking on a beach or task intentions. Thus, the effect of II might have been attenuated because we have included more comparable standardized instructions for the control condition.

Evidence suggests that II should strengthen the commitment to completing a PM task, which results in greater importance being placed on its successful execution (Kliegel et al., 2001, 2004) or strengthen the cue-response link to facilitate successful prospective remembering. Therefore, if a strong commitment was made to enrich encoding, then a cost to the ongoing task for participants in the II condition would be anticipated (i.e., slower response times to the ongoing task, fewer correct responses, or both). However, none of these patterns in performance were observed. Both CI and II groups performed similarly on the ongoing task in their respective age groups. That is, participants in CI and II conditions responded on average, at the same speed, and with similar accuracy within their own age group. We only observed the age effect whereby older adults performed worse in terms of accuracy and slower response latencies compared to younger adults. These findings are also contrary to the multi-process framework Scullin et al., 2013)'s prediction that II should facilitate performance for tasks that require strategic monitoring (i.e., in the non-focal event-based tasks). Taken together, these nonsignificant findings may indicate that a combined visual and verbal II strategy is not sufficient to improve PM in our non-focal event-based laboratory-based task in high functioning samples.

### Transfer

No near- or far-transfer effects were observed for either age group. To the best of our knowledge, this is the first study that has investigated the effects of II using a time-delay between the sessions. The hypotheses were guided by research on healthy young adults and older adults demonstrating a facilitation effect of II on PM performances that was significant with a medium and medium-to-large effect size, respectively (Chen et al., 2015). Despite means trending in the predicted direction for older adults, the II facilitation effect did not transfer to the second laboratory-based PM task. This is somewhat congruent with other studies that show the effectiveness of II on older adults' PM but find no reliable far-transfer effects to other cognitive domains (Chasteen et al., 2001; Liu and Park, 2004; Schnitzspahn and Kliegel, 2009; McFarland and Glisky, 2011; Bugg et al., 2013; Brom and Kliegel, 2014; Lee et al., 2016; Shelton et al., 2016; Henry et al., 2020). An explanation for the lack of far-transfer could be due to the different cue focality in the ecological task. Within the ecological task, the event-based PM cue was focal and the time-based PM was non-focal cue while there was only a non-focal PM cue in the laboratory-based PM task. Although it would be ideal to compare non-focal event-based also in this ecological task, the nature of a time-based task is almost always non-focal. This task was designed to simulate daily life, thus, is still appropriate for a far-transfer category.

Other studies that have found lasting effects in PM performance and significant transfer effects to functional capacity are mostly within subclinical populations (e.g., Burkard et al., 2014a; Waldum et al., 2016). Other research has found that poor planning, not using external aids to their full advantage, and having divided attention while encoding can negatively impact PM performance (Einstein et al., 1997; Shum et al., 2013). Some have concluded that training effects may be attributed to the emphasis on giving explicit instructions and encouraging participants to practice the learned techniques at home and discussing the experience in using these strategies regularly (McDaniel et al., 2014; Waldum et al., 2016). Henry et al. (2021) found that PM interventions that target a single cognitive ability do not reliably generate large far-transfer effects in healthy populations. They suggested that improving older adults' PM functioning using an integrated training approach (both restorative and compensatory approaches) is more effective than targeting a single cognitive ability. Thus, using external aids and restorative approaches (e.g., repetition and rehearsal) to target multiple cognitive domains relevant to PM may have more potential to enhance far-transfer.

### Implications

These findings add to the current body of literature aiming to understand of the parameters in which II is an effective strategy in improving PM performance. These findings suggest that repeated encoding instructions are required to facilitate the strength of future intentions during the formation phase of the PM process, rather than single-session II. Thus, follow-up support and feedback on the use of II in everyday life is essential to promote skills transfer. This is important when investigating PM in an aging population as the goal of PM research is to improve daily functioning. The non-significant effects of II for the high functioning sample in this study, may suggest that metacognitive strategies are not effective for those with such characteristics. It is important to explore transfer effects between laboratory and ecological everyday tasks because there are limited studies examining this aspect for PM interventions.

### Limitations and future research

Key limitations of this study relate to the sample size and lack of matching of the young adults and older adults on education and cognitive ability. However, controlling for these variables did not affect the outcomes. These non-significant findings for II training may be due to the insufficient sample size, and hence lack of statistical power to detect between-group differences. However, power analyses revealed that a total sample of 124 was required for a medium sized effect, which was comparable to our sample size obtained. In addition, the nonsignificant finding may also be due to the more comparable evaluation of the II and CI conditions. It is also important to acknowledge that younger adults performed very well on the laboratory-based PM tasks, and this was true regardless of condition. Consequently, the main determinant of age-related effects in the present study was variability in older adults' performance. Our results also caution against the transfer effects of this strategy onto other PM tasks. That is, the single and brief training session for II does not appear sufficient to elicit a transfer effects onto a latter task, especially among a healthy high functioning population. Participants may need practice and explicit reminders of the strategy prior each PM task. Rehabilitation studies have shown that using a technique only once with a patient or simply explaining its use is insufficient to guarantee transfer or applications in real-life situations (McDaniel and Bugg, 2012; Burkard et al., 2014a). Moreover, the higher functioning sample of older adults, limits the generalizability of these findings. Thus, future studies should recruit more diverse groups of older and young adults to further examine the effectiveness and benefits of II. Moreover, given the ubiquity of PM tasks in daily life, future work should examine the effects of II on PM using other naturalistic tasks that more closely reflect the requirements in older adults' daily lives, considering the need for experimental control.

## Conclusion

While the findings from this study did not support the overall facilitation effect of II on PM performance or transfer effects, strong age effects were consistently observed on the laboratory and ecological PM tasks. These findings suggest that brief training in the use of II may not enhance PM performance for adults with high functioning. Repeated encoding of II may be required to elicit a facilitation effect, although this remains to be investigated.

## Data availability statement

The raw data supporting the conclusions of this article will be made available by the authors, without undue reservation.

## Ethics statement

The studies involving human participants were reviewed and approved by Griffith University. The patients/participants provided their written informed consent to participate in this study.

## Author contributions

YK and DS analyzed the data. YK wrote the manuscript. All authors provided critical revisions to the manuscript and contributed to the conception of the work.

## Funding

DS was supported by the Yeung Tsang, Wing Yee, and Tsang Wing Hing Endowed Professorship.

## Conflict of interest

The authors declare that the research was conducted in the absence of any commercial or financial relationships that could be construed as a potential conflict of interest.

## Publisher's note

All claims expressed in this article are solely those of the authors and do not necessarily represent those of their affiliated organizations, or those of the publisher, the editors and the reviewers. Any product that may be evaluated in this article, or claim that may be made by its manufacturer, is not guaranteed or endorsed by the publisher.

## Footnotes

^1^ANCOVAs were also conducted including WM, FSIQ, and years of education for all analyzes, however this did not affect the outcome (all *p*s > 0.05). That is, there was no main effect of condition or significant interactions across all tasks. Only main effect of age group was significant.
